# Transdiagnostic processes in university mental health: the role of psychological inflexibility, impulsivity and stress in depression and life satisfaction using structural equation modeling

**DOI:** 10.3389/fpubh.2026.1924538

**Published:** 2026-08-27

**Authors:** María Paladines-Costa, Víctor López-Guerra, Encarnación Sarriá, Patricia Recio, Pablo Torres-Carrión

**Affiliations:** 1 Department of Methodology of Behavioral Sciences, Faculty of Psychology, Universidad Nacional de Estudios a Distancia (UNED), Madrid, Spain; 2 Department of Psychology, Faculty of Behavioral Sciences, Universidad Técnica Particular de Loja (UTPL), Loja, Ecuador; 3 Department of Computer Science, Faculty of Engineering and Architecture, Universidad Técnica Particular de Loja, Loja, Ecuador

**Keywords:** depression, impulsivity, life satisfaction, psychological inflexibility, perceived stress, public mental health, university students

## Abstract

**Background:**

Perceived stress is a central transdiagnostic process in university mental health, yet the mechanisms linking dispositional vulnerabilities to psychological distress and positive wellbeing remain insufficiently understood. This study examined whether perceived stress mediates the associations of psychological inflexibility and impulsivity with depressive symptoms and life satisfaction among Ecuadorian university students.

**Methods:**

This cross-sectional study included 4,524 Ecuadorian university students. Psychological inflexibility, impulsivity, perceived stress, depressive symptoms, and life satisfaction were assessed using validated self-report measures. A theoretically specified structural equation model tested perceived stress as a mediator between psychological inflexibility and impulsivity and both mental health outcomes. Indirect effects were estimated using bias-corrected bootstrapping with 5,000 resamples.

**Results:**

The model showed adequate overall fit, χ^2^(1) = 27.469, *p* < 0.001, CFI = 0.997, NFI = 0.997, SRMR = 0.0153, and RMSEA = 0.076, 90% CI [.053,0.102]. Psychological inflexibility was associated with perceived stress (β = 0.587), depressive symptoms (β = 0.475), and life satisfaction (β = −0.261). Impulsivity was associated with perceived stress (β =0.143) and depressive symptoms (β = 0.097). Perceived stress was associated with depressive symptoms (β = 0.290) and life satisfaction (β = −0.371). Significant indirect effects through perceived stress were found for psychological inflexibility on depressive symptoms [β = 0.170, 95% BC CI (153, 0.189)] and life satisfaction [β = −0.218, 95% BC CI (−0.240, −0.197)], and for impulsivity on depressive symptoms [β =0.041, 95% BC CI (033, 0.050)] and life satisfaction [β = −0.053, 95% BC CI (−0.065, −0.042)]. The model explained 55% of depressive symptoms and 33% of life satisfaction variance.

**Conclusion:**

Perceived stress emerged as a central mechanism linking psychological inflexibility and impulsivity with distress- and wellbeing-related outcomes. Psychological inflexibility showed stronger direct and indirect associations, whereas impulsivity showed smaller effects primarily through perceived stress. These findings identify potentially relevant transdiagnostic targets for university mental health promotion and prevention. Given the cross-sectional design, indirect pathways should not be interpreted as temporal or causal effects and require longitudinal confirmation.

## 1 Introduction

Mental health is increasingly understood as a multidimensional construct that encompasses both negative and positive aspects of psychological functioning, including psychological distress and subjective wellbeing. Within this framework, depression and life satisfaction represent related yet partially independent indicators of mental health, reflecting psychological difficulties and positive functioning, respectively. From a public health perspective, examining the psychological processes that simultaneously influence these outcomes is essential for informing strategies aimed at preventing mental health problems while promoting wellbeing at the population level.

Depression is a common mental disorder characterized by the persistent presence of depressed mood and loss of interest or pleasure, affecting approximately 280 million people worldwide, around 3.8% of the global population, with an estimated prevalence of 5% among adults (1). Beyond its clinical manifestations, it represents a substantial contribution to the global burden of disease, adversely affecting quality of life and significantly increasing mortality and disability rates (2, 3). In the Region of the Americas, depression accounts for 7.8% of years lived with disability, increasing to 8.3% in Ecuador, placing the country among the five most affected in Latin America (4). University students constitute a particularly vulnerable population: more than one quarter (28.9%) of Latin American and U.S. university students present depressive symptoms that warrant clinical treatment (5), and clinically significant levels have been consistently documented in Latin American university samples (6–10), making depression in higher education a major public mental health concern.

Life satisfaction is defined as a cognitive evaluation of one's overall life circumstances and constitutes a core component of subjective wellbeing (11, 12), playing a fundamental role in emotional, psychological, and social development (13, 14). Among Ecuadorian university students, 38.9% present moderate levels of life satisfaction (15), and other studies indicate that levels in university populations often range from moderate to low, increasing vulnerability to mental health problems (16–18). Evidence consistently shows that life satisfaction acts as a protective factor against depressive symptomatology, with a strong negative association reported between the two constructs (19) and comparable correlations documented among university students (20–22). These results support a dual-factor conceptualization of mental health, in which depressive symptomatology and wellbeing constitute related yet partially independent dimensions: reducing psychological distress does not necessarily imply the presence of wellbeing, and vice versa. Although life satisfaction has frequently been examined as a protective factor against psychological distress, in the present study it is not modeled in that role. Consistent with the dual-factor perspective, life satisfaction is specified as an outcome variable, that is, as an indicator of positive mental health that, alongside depressive symptomatology, is influenced by the transdiagnostic processes under examination. The protective evidence reviewed above is therefore treated as background justifying its inclusion as a distinct outcome, and not as a hypothesis about its predictive role.

Prior research has also documented that life satisfaction plays a protective role in stressful situations by facilitating acceptance and adaptive coping (19, 23); however, subjective wellbeing may be compromised by the excessive external demands university students face, such as academic pressure and the transition to independent adulthood (24–30). These pressures negatively affect mental health, academic performance, and social functioning, thereby increasing perceived stress among students (31–36). Illustratively, 68.55% of U.S. university students experience severe stress (37), while in Ecuador perceived stress levels range from moderate to high (38, 39).

In this context, the transdiagnostic approach offers a valuable framework for public mental health research by focusing on psychological processes that cut across diagnostic categories and influence multiple mental health outcomes simultaneously. Prior research suggests that at least three transdiagnostic variables are involved in depression and reduced wellbeing. First, perceived stress is defined as the perceived inability to cope with environmental demands: according to stress appraisal theories, it reflects individuals' perceptions that environmental demands exceed their available coping resources, generating emotional and cognitive responses that may compromise psychological adjustment (40–42). Perceived stress has been examined in two conceptually distinct roles. As a predictor, it accounts directly for variance in mental health outcomes, and has been consistently identified as a major risk factor and a key determinant of depression (43, 44), with higher levels associated with increased psychological distress, particularly depressive and anxiety symptoms (45). As a mediator, it transmits the influence of more distal individual characteristics: dispositional vulnerabilities such as psychological inflexibility or deficits in self-regulation may increase the tendency to appraise daily situations as uncontrollable or overwhelming, thereby elevating perceived stress and subsequently contributing to poorer psychological adjustment, a pathway supported by empirical mediation evidence (46, 47). These roles are not mutually exclusive. In the present model, perceived stress is specified simultaneously in both: as the proximal mechanism through which psychological inflexibility and impulsivity are associated with the two outcomes, and as a direct predictor of depressive symptomatology and life satisfaction.

Second, psychological inflexibility refers to a behavioral regulation process characterized by the tendency to avoid or escape aversive internal experiences, even when such avoidance leads to behavior that is inconsistent with personal values and goals (41, 48). This process interferes with adaptive coping and adjustment to stress, undermining emotional wellbeing and contributing to depressive symptoms (49, 50). Its transdiagnostic status derives from the fact that it describes how individuals respond to aversive internal experiences rather than the diagnostic content or form of those experiences, so that the same avoidance repertoire operates across symptom domains rather than being specific to any one of them. This process is particularly salient in university populations: the academic and evaluative demands described above generate recurrent aversive thoughts and emotions, and attempts to suppress or avoid them compete directly with the sustained, value-consistent behavior that academic progress requires, which links inflexibility both to distress and to diminished wellbeing in this population (48, 51–53).

Third, impulsivity is characterized by difficulties in self-regulation and inhibitory control and has been associated with a wide range of mental health and social problems, including depression (54, 55). Two complementary pathways may account for its relevance to the outcomes examined here. First, deficient inhibitory control increases the frequency of poorly regulated responses to everyday demands and their adverse consequences, which may lead individuals to appraise those demands as uncontrollable and thereby elevate perceived stress, consistent with evidence identifying impulsivity as a predictor of both depressive symptoms and perceived stress (56, 57). Second, a tendency to act without regard to longer-term consequences interferes with the sustained goal-directed behavior on which academic progress depends, and thus with the cognitive appraisal of one's life circumstances that defines life satisfaction (11, 12). Positive associations between impulsivity and depressive symptomatology have been consistently reported in student samples (58–60).

Empirical evidence in university populations converges on three findings. First, perceived stress shows the most robust and consistent associations: it correlates positively with depressive symptomatology among Ecuadorian students (39) and across a wide range of samples (58, 61–67), and it has also been identified as a predictor of depression and of lower life satisfaction, which is in turn predicted by lower levels of depression and stress (68), with additional evidence linking increased stress to reduced life satisfaction (69, 70). Second, psychological inflexibility is positively associated with depression (48) and predicts depressive symptoms in students (51–53), while relating negatively to life satisfaction, which is itself inversely associated with both inflexibility and stress (71). Third, impulsivity displays a comparable but less extensively documented pattern (56–60). Taken together, the three processes have been shown to predict both depression and life satisfaction in university populations (6, 72–74).

A further consideration concerns how these variables have been ordered in previous mediation models. In Ecuadorian samples, perceived stress and psychological inflexibility jointly predicted depressed mood, but inflexibility was specified as the mediator of the effect of stress on depressive symptoms (45); a comparable specification identified stress as the strongest predictor of depression, again with inflexibility mediating its impact on psychopathology (41). The present study examines the complementary specification, in which the dispositional processes are treated as distal vulnerabilities and perceived stress as the proximal mechanism, consistent with appraisal theories in which stable individual characteristics shape how environmental demands are evaluated (40–42).

**Figure 1:**
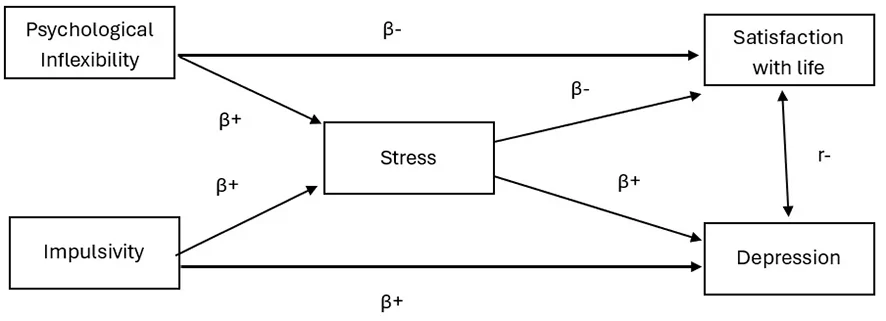
Hypothesized theoretical model of the relationships between psychological inflexibility, impulsivity, perceived stress, and mental health outcomes. β+ and β- indicate the direction of the hypothesized associations (positive and negative, respectively); r– indicates a hypothesized negative covariance between the two outcomes. Psychological inflexibility and impulsivity are specified as distal dispositional vulnerabilities, and perceived stress as the proximal mechanism through which they are hypothesized to relate to depressive symptoms and life satisfaction. No direct path from impulsivity to life satisfaction is specified.

Taken together, the evidence indicates that perceived stress, psychological inflexibility, and impulsivity constitute key transdiagnostic processes for understanding both depressive symptomatology and life satisfaction in university students (see Figure 1). However, there remains a need to examine their interactions within integrated models, particularly in Latin American contexts where stress and depressive symptoms are highly prevalent. Because these are modifiable determinants, clarifying such mechanisms is essential for developing scalable and cost-effective public mental health strategies aimed not only at reducing the burden of depression, but also at promoting life satisfaction among university students. Based on the proposed theoretical model (Figure 1), the present study examines the mediating role of perceived stress in the relationships between psychological inflexibility, impulsivity, depression, and life satisfaction among Ecuadorian university students. Accordingly, perceived stress is expected to account for significant indirect associations between both dispositional vulnerability factors and the two mental health outcomes, while direct associations are expected to remain for the pathways specified in the proposed structural model. Consequently, the following hypotheses are proposed:

H1: Psychological inflexibility and impulsivity are positively associated with perceived stress;

H2: Perceived stress positively predicts depressive symptomatology and negatively predicts life satisfaction;

H3: Perceived stress mediates the associations of psychological inflexibility and impulsivity with depressive symptomatology and life satisfaction.

H4: Psychological inflexibility retains significant direct associations with both outcomes, whereas impulsivity retains a significant direct association with depressive symptoms after accounting for perceived stress.

## 2 Materials and methods

### 2.1 Participants

The participants were undergraduate students enrolled at three universities in Ecuador. A non-probabilistic, non-clinical convenience sample was used. The initial sample consisted of 5,996 students. A total of 1,472 questionnaires were excluded because they contained incomplete responses on one or more of the study measures, preventing the computation of complete scale scores required for the statistical analyses. The final analytical sample comprised 4,524 students.

Of the total sample, 55% were women. The mean age of the participants was 21.74 years (SD = 3.15), with ages ranging from 17 to 58 years. Most participants were single (94%), and the majority were enrolled as full-time students (90%).

Regarding ethnic self-identification, most participants identified as mestizo (90%), followed by Indigenous (5%), Afro-Ecuadorian (2%), and White (1.2%). Students were recruited from three higher education institutions: the Technical University of the North, the Technical Private University of Loja, and the Salesian Polytechnic University.

Participation was entirely voluntary, and no financial or academic compensation was provided. All respondents were enrolled as in-person undergraduate students at the time of data collection.

Due to the non-probabilistic convenience sampling strategy, the sample is not representative of the Ecuadorian university population. Therefore, the findings should be interpreted with caution, and their generalizability is primarily limited to university populations with characteristics similar to those included in the present study.

### 2.2 Measures

#### 2.2.1 Sociodemographic Data

This section collected basic sociodemographic information, including participants' age, sex, marital status, and geographic region of residence.

#### 2.2.2 Satisfaction with Life Scale (SWLS)

Life satisfaction was assessed using the SWLS, originally developed by Diener et al. (1985). The SWLS is a self-report instrument designed to measure global cognitive judgments of satisfaction with one's life as a whole.

The scale consists of five items rated on a 7-point Likert-type scale ranging from 1 (“completely disagree”) to 7 (“completely agree”). Total scores range from 5 to 35, with higher scores indicating greater life satisfaction. An example item is: “*In most ways my life is close to my ideal”*.

The SWLS was adapted and validated for use with Ecuadorian university students by López-Guerra et al. (2024). The Ecuadorian version corresponds to the Spanish adaptation of the SWLS, which was psychometrically validated for use with Ecuadorian university students and was administered in its validated form without further modifications. In this population, the scale demonstrated a robust unidimensional structure with excellent model fit indices and full factorial invariance across sex. Internal consistency was satisfactory (ω = 0.84), and the scale showed strong negative correlations with depressive symptoms and perceived stress, supporting adequate divergent validity. In the present study, the SWLS showed good internal consistency (Cronbach's α = 0.87).

#### 2.2.3 Patient health questionnaire-9 (PHQ-9)

Depressive symptoms were assessed using the PHQ-9, originally developed by Kroenke et al. (75). The PHQ-9 is a self-report measure designed to assess depressive symptomatology according to DSM-IV criteria over the previous 2 weeks.

The instrument consists of 9 items rated on a 4-point Likert-type scale ranging from 0 (“not at all”) to 3 (“nearly every day”). Total scores range from 0 to 27, with higher scores indicating greater severity of depressive symptoms.

The PHQ-9 was adapted and psychometrically validated in Ecuadorian university students by López-Guerra et al. (6). This validation study reported adequate internal consistency (ω = 0.90) and a hierarchical factor structure including one general factor and three specific factors (somatic, cognitive/affective, and concentration/motor). The scale also demonstrated satisfactory convergent and divergent validity with multiple health indicators. In the present study, the PHQ-9 showed high internal consistency (Cronbach's α = 0.91). Although the Ecuadorian validation supported a hierarchical structure comprising one general factor and three specific factors (somatic, cognitive/affective, and concentration/motor), only the total PHQ-9 score was used in the present study to represent overall depressive symptom severity.

#### 2.2.4 Perceived stress scale (PSS-10)

Perceived stress was evaluated using the PSS-10, originally developed by Cohen et al. (42). The PSS-10 is a self-report instrument that assesses the degree to which individuals perceive situations in their lives as stressful, unpredictable, and uncontrollable during the previous month.

The scale consists of 10 items rated on a 5-point Likert-type scale ranging from 0 (“never”) to 4 (“very often”). Items 4, 5, 7, and 8 are reverse scored prior to computing the total score. Reverse-scored items were recoded according to the original scoring instructions before calculating the total scores. Total scores range from 0 to 40, with higher scores indicating higher levels of perceived psychological stress.

The PSS-10 was adapted and validated for the Ecuadorian context by Ruisoto et al. (39). This study reported good internal consistency (α = 0.85; ω = 0.87), a bifactorial structure explaining 56.99% of the total variance, and satisfactory convergent validity with multiple health-related indicators. In the present study, the scale also demonstrated good internal consistency (Cronbach's α = 0.83).

#### 2.2.5 Acceptance and commitment questionnaire-II (AAQ-II)

Psychological inflexibility was assessed using the AAQ-II, originally developed by Bond et al. (76). The AAQ-II is the most widely used self-report instrument for assessing psychological inflexibility, a core transdiagnostic process characterized by rigid behavioral patterns driven by experiential avoidance and attempts to control aversive internal experiences.

The questionnaire consists of seven items rated on a 7-point Likert-type scale ranging from 1 (“never”) to 7 (“always”). Total scores range from 7 to 49, with higher scores indicating greater psychological inflexibility and a stronger tendency to avoid or control unpleasant thoughts, emotions, and memories. An example item is: “*I worry about not being able to control my worries and feelings.”*

The AAQ-II was linguistically and culturally adapted and psychometrically validated for Ecuadorian university students by Paladines-Costa et al. (48). In this validation study, conducted with a large sample of Ecuadorian students, the scale demonstrated a robust unidimensional factor structure explaining between 66.87% and 70.03% of the total variance, with excellent model fit indices (CFI = 0.995, GFI = 0.992, SRMR = 0.037, RMSEA = 0.047).

Regarding reliability, the Ecuadorian version of the AAQ-II showed optimal internal consistency (Cronbach's α = 0.919; McDonald's ω = 0.928) and satisfactory convergent and divergent validity, with significant associations with perceived stress, depressive and anxiety symptoms, loneliness, resilience, and quality of life indicators (48). In the present study, the AAQ-II also demonstrated excellent internal consistency (Cronbach's α = 0.92). The instrument was used according to the previously validated Ecuadorian version, which supports a unidimensional factor structure (48).

#### 2.2.6 Barratt Impulsiveness scale—brief (BIS-8)

Impulsivity was assessed using the BIS-8, developed by Steinberg et al. (77) as a shortened version of the original 30-item Barratt Impulsiveness Scale. The BIS-8 is a self-report instrument designed to assess general impulsivity as a stable personality trait related to rapid, unplanned reactions and deficits in self-regulation and inhibitory control.

The scale consists of eight items rated on a 4-point Likert-type scale ranging from 1 (“rarely/never”) to 4 (“almost always/always”). Total scores are obtained by summing item responses, with higher scores indicating higher levels of impulsivity. An example item is: “*I act on the spur of the moment.”*

The BIS-8 was developed using item response theory analyses, resulting in a unidimensional structure that retains the strongest indicators of the impulsivity construct while substantially reducing respondent burden. The original validation study demonstrated adequate internal consistency (Cronbach's α ≈ 0.80) and strong correlations with the full BIS-11 total score, supporting its construct validity across community, clinical, and forensic samples (77). The BIS-8 has been recommended for use in large-scale epidemiological and mental health studies due to its efficiency and solid psychometric performance. In the present study, the BIS-8 showed adequate internal consistency for a brief instrument (Cronbach's α =0.69).

### 2.3 Design and procedure

This study employed a nonexperimental, cross-sectional design aimed at examining the associations among the variables included in the proposed theoretical model, without direct manipulation or experimental control (78). The methodological approach was grounded in structural equation modeling (SEM) to test the hypothesized relationships between the psychological constructs under study.

The research was conducted as part of a broader project focused on predicting substance use among university students from three higher education institutions in Ecuador. Data collection was carried out through an online survey, which facilitated access to a large student population across institutions. Undergraduate students were initially invited to participate via institutional email. Subsequently, a 10-week awareness and dissemination campaign was implemented using internal university communication channels, official social media platforms, and on-campus informational activities at the three participating universities. These strategies aimed to increase student engagement and voluntary participation in the study.

The administration of the assessment instruments was conducted entirely online. The average response rate across universities was 47.8%, with institutional rates ranging from 39.1% to 56.1%, reflecting acceptable participation levels for large-scale online surveys in academic settings.

The study protocol was reviewed and approved by the Human Research Ethics Committee (Comité de Ética de Investigación en Seres Humanos, CEISH) of the Universidad Técnica Particular de Loja (UTPL), Ecuador (approval code: UTPL-DIS-2019-0088-O, March 6, 2019). All procedures were conducted in accordance with the ethical principles outlined in the Declaration of Helsinki (79).

Prior to participation, all students provided digital informed consent, confirming their voluntary participation and understanding of the study objectives. Participants were assured of the confidentiality and anonymity of their responses. Upon completion of the assessment, participants received personalized feedback regarding their results as part of the ethical commitment to transparency and participant benefit.

### 2.4 . Statistical analyses

Data analyses were conducted using IBM SPSS Statistics version 27 (IBM Corp., Armonk, NY, USA). In an initial step, descriptive statistics were computed for all study variables, including measures of central tendency (mean), dispersion (minimum, maximum, standard deviation, and coefficient of variation), and distribution shape (skewness and kurtosis). Questionnaires with incomplete responses on one or more study measures were excluded prior to the analyses. Consequently, all descriptive analyses and the structural equation model were estimated using a complete-case (listwise deletion) approach based on the final analytical sample of 4,524 participants, and no imputation procedures were applied. Given the large final sample size and the objective of estimating the structural paths among the study variables using complete scale scores, the complete-case approach was considered appropriate. Nevertheless, the exclusion of incomplete questionnaires may have introduced some degree of selection bias, and its potential influence on the parameter estimates cannot be entirely ruled out. This issue is therefore acknowledged as a study limitation.

Univariate normality was examined using skewness and kurtosis coefficients, adopting absolute values lower than 2 for skewness and 7 for kurtosis as acceptable thresholds (80). Additionally, the Kolmogorov–Smirnov and Shapiro–Wilk tests were applied, yielding statistically significant results for all variables (*p* < 0.001), a common outcome in large samples. Given the large sample size (*n* = 4,524) and the fact that skewness and kurtosis values remained within acceptable ranges, the variables were assumed to approximate normal distributions for analytical purposes (81).

In a second step, bivariate associations among the study variables were examined using Pearson correlation coefficients, with statistical significance set at *p* < 0.05. The magnitude of correlations was interpreted following Cohen's criteria (42), whereby values of approximately 0.10 indicate small effects, 0.30 moderate effects, and values equal to or greater than 0.50 large effects.

In a third step, a mediation model was tested using structural equation modeling (SEM) with Maximum Likelihood (ML) estimation implemented in AMOS version 25 (Arbuckle, 2017). To enhance the robustness of the mediation analysis, bootstrapping with 5,000 resamples was employed to estimate the significance of indirect effects through bias-corrected confidence intervals. The structural model was specified *a priori* based on the theoretical framework and previous empirical evidence; therefore, the objective was to evaluate the fit of the hypothesized model rather than to compare competing structural models.

Potential multicollinearity among the predictor variables (psychological inflexibility, impulsivity, and perceived stress) was assessed using Pearson correlation coefficients. All intercorrelations were below the conventional cut-off of 0.80 (82), with the highest value observed between psychological inflexibility and perceived stress (r = 0.671). Multicollinearity was therefore considered non-problematic.

The overall fit of the structural model was evaluated using multiple goodness-of-fit indices in accordance with established recommendations (83), including the chi-square statistic (χ^2^) and its associated degrees of freedom and *p* value; the Comparative Fit Index (CFI) and the Normed Fit Index (NFI), with values greater than.90 considered acceptable and greater than.95 indicative of excellent fit; the Standardized Root Mean Square Residual (SRMR), with values below.08 reflecting acceptable fit and below.05 good fit; and the Root Mean Square Error of Approximation (RMSEA), with values below.08 interpreted as acceptable and below.05 as excellent. Given the sensitivity of the chi-square statistic to large sample sizes, model fit was evaluated based on the overall pattern of complementary fit indices rather than on the chi-square test alone.

## 3 Results

### 3.1 Descriptive analysis

Table 1 presents the descriptive statistics for life satisfaction. Students obtained a mean score of 24.29 out of a maximum of 35 points, which, according to the normative criteria proposed by Pavot and Diener (84), reflects a generally positive level of life satisfaction. The standard deviation (SD = 6.33) and coefficient of variation (CV = 26.6%) indicate moderate variability among participants, suggesting relevant individual differences in perceived wellbeing. Regarding distribution shape, a slight negative skewness (Sk = −0.484) was observed, indicating a tendency toward higher scores on the scale, while the slightly negative kurtosis (K = −0.164) suggests a mildly flattened distribution with moderate dispersion around the mean.

**Table 1:** Descriptive statistics and reliability coefficients of the study variables.

Variables	*M*	*SD*	CV	AS	K	Min.	Max.
1. Satisfaction with life	24.29	6.33	26.6%	−0.484	–*0.164*	5	35
2. Depression	6,42	5,76	89.72%	1.23	*1.27*	0	27
3. Stress	13,42	5,04	37.56%	0.174	0.156	0	40
4.Psychological inflexibility	21,41	10,34	48.40%	0.608	−0.384	7	49
5. Impulsivity	18.68	2.80	14.99%	0.601	0.124	9	29

M, arithmetic mean; SD, standard deviation; CV, coefficient of variation; AS, coefficient of skewness; K, coefficient of kurtosis; Min, minimum values, Max, maximum values.

With respect to depressive symptoms, students obtained a mean score of 6.42 out of a maximum of 27 points, which corresponds to mild symptomatology according to the cut-off criteria proposed by Kroenke et al. (75). The coefficient of variation was high (CV = 89.72%), reflecting substantial variability across participants. The distribution showed a pronounced positive skewness (Sk = 1.23), indicating that most students reported low symptom levels, accompanied by a smaller proportion of higher scores. In addition, the positive kurtosis (K = 1.27) indicates a leptokurtic distribution, suggesting greater concentration of scores around the mean and heavier tails.

For perceived stress, students obtained a mean score of 13.42 out of a possible 40 points, indicating a low level of stress based on commonly used criteria for this scale. The standard deviation (SD = 5.04) and coefficient of variation (CV = 37.56%) reflect moderate dispersion and heterogeneity in perceived stress levels. The distribution was close to symmetry, with a slight tendency toward lower values, while the kurtosis close to zero (K = 0.156) indicates a distribution very similar to normal.

Regarding psychological inflexibility, students obtained a mean score of 21.41 (SD = 10.34) out of a maximum of 49 points, representing a moderate level of this construct and falling below the clinical cut-off score of 29 proposed by Suárez-Falcón et al. (85). The relatively large standard deviation and coefficient of variation (CV = 48.40%) indicate high variability within the sample. The distribution showed moderate positive skewness (Sk = 0.608), suggesting a higher concentration of scores at lower levels, while the negative kurtosis (K = −0.384) indicates a flatter-than-normal distribution.

In relation to impulsivity, students obtained a mean score of 18.68 (SD = 2.80) on a scale with a theoretical range of 8–32; the observed scores in the present sample ranged from 9 to 29. The low coefficient of variation (CV = 14.99%) reflects low dispersion and high homogeneity across participants. The distribution exhibited slight positive skewness (Sk = 0.601) and kurtosis close to zero (K = 0.124), indicating a shape close to normality.

Overall, the descriptive analyses indicate that students generally report high life satisfaction and low levels of depressive symptoms and perceived stress, while psychological inflexibility and impulsivity fall within moderate, non-clinical ranges. Nevertheless, the substantial variability observed in depressive symptoms and psychological inflexibility suggests the presence of meaningful individual differences, supporting the examination of explanatory mechanisms among these variables in subsequent analyses.

### 3.2 Bivariate correlations

Table 2 presents the bivariate correlations among life satisfaction, depression, stress, psychological inflexibility, and impulsivity. All correlations were statistically significant (*p* < 0.001), indicating consistent associations among the variables included in the analysis.

**Table 2:** Pearson correlation coefficients among study variables.

Variables	1	2	3	4	5
1. Satisfaction with life	1	−0.505^*^	0.525^*^	−0.498^*^	−0.285^*^
2. Depression		1	0.646^*^	0.695^*^	0.379^*^
3. Stress			1	0.671^*^	0.363^*^
4. Psychological inflexibility				1	0.363^*^
5.Impulsivity					1

^*^p < 0.001 (two-tailed). All correlations are statistically significant.

Life satisfaction was negatively associated with depression (r = −0.505), stress (r = −0.525), psychological inflexibility (r = −0.498), and impulsivity (r = −0.285), suggesting that higher levels of subjective wellbeing are linked to lower psychological distress and fewer impulsive tendencies. Depression showed positive correlations with stress (r = 0.646), psychological inflexibility (r = 0.695), and impulsivity (r = 0.379), indicating that greater depressive symptomatology co-occurs with increased emotional distress, reduced flexibility, and higher impulsivity.

Stress was positively correlated with psychological inflexibility (r = 0.671) and impulsivity (r = 0.363), reflecting the concurrence of perceived stress with difficulties in emotional regulation and attentional control. Psychological inflexibility exhibited a central position within the correlational pattern, showing negative associations with life satisfaction (r = −0.498) and positive associations with depression (r = 0.695), stress (r = 0.671), and impulsivity (r = 0.363). Finally, impulsivity was positively correlated with depression (r = 0.379), stress (r = 0.363), and psychological inflexibility (r = 0.363), and negatively correlated with life satisfaction (r = −0.285).

Importantly, although several correlations were of low to moderate magnitude, none approached levels typically considered indicative of severe multicollinearity among observed variables (e.g., r ≥0.80). This pattern suggests that the constructs, while related, retain sufficient empirical distinctiveness. Accordingly, the correlation matrix supports the suitability of the data for subsequent path analysis within a structural equation modeling framework, enabling the examination of simultaneous relationships among the observed variables.

### 3.3 Structural equation model analysis

**Figure 2:**
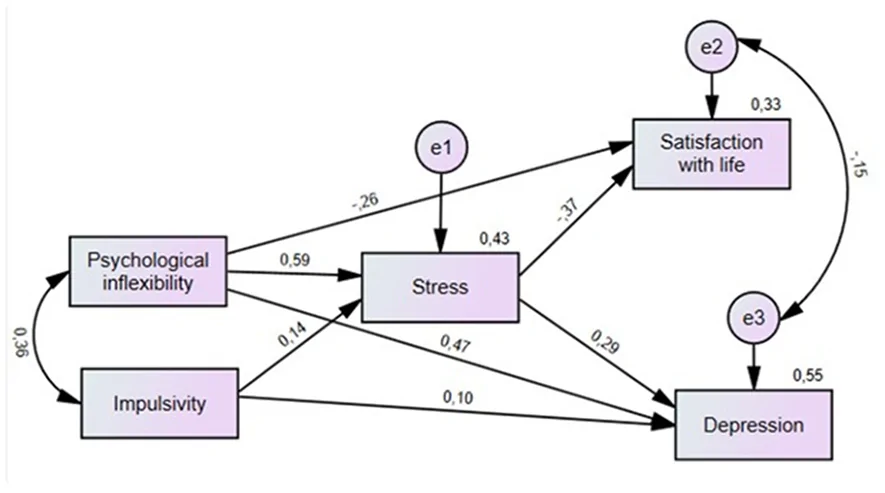
Structural model of the relationships between psychological inflexibility, impulsivity, perceived stress, and mental health outcomes. *N* = 4,524. Values are standardized path coefficients (β). Single-headed arrows represent directional paths; double-headed arrows represent covariances. The double-headed arrow between psychological inflexibility and impulsivity represents the covariance between the two exogenous predictors (r = 0.363). The double-headed arrow between depressive symptoms and life satisfaction represents the covariance between their disturbance terms (r = −0.15), that is, the residual association remaining after accounting for the predictors in the model; it is not comparable to the bivariate correlation reported in Table 2 (r = −0.505). R^2^ values indicate the proportion of variance explained in each endogenous variable: perceived stress (R^2^ = 0.43), depressive symptoms (R^2^ = 0.55), and life satisfaction (R^2^ = 0.33). A direct path from impulsivity to life satisfaction was not specified in the model. All estimated paths were statistically significant (*p* < 0.001). Model fit: χ^2^(1) = 27.469, *p* < 0.001; CFI = 0.997; NFI = 0.997; SRMR = 0.0153; RMSEA = 0.076, 90% CI [0.053, 0.102].

A structural equation model (SEM) was estimated to test the hypothesized structural model linking psychological inflexibility, impulsivity, perceived stress, life satisfaction, and depression (see Figure 2). Consistent with the study hypotheses, the model was specified *a priori* based on the proposed theoretical framework and previous empirical evidence. Accordingly, the objective was to evaluate the fit of the hypothesized structural model rather than to compare competing structural models. The model demonstrated an adequate overall fit to the data, χ^2^(1) = 27.469, *p* < 0.001, CFI = 0.997, NFI = 0.997, SRMR = 0.0153, and RMSEA = 0.076, 90% CI [0.053, 0.102].

Psychological inflexibility emerged as a strong predictor of perceived stress (β = 0.59) and showed significant direct effects on depression (β = 0.47) and life satisfaction (β = −0.26). In contrast, impulsivity was a significant predictor of both perceived stress (β = 0.14) and depression (β = 0.10), although with smaller effect sizes. A positive covariance was observed between psychological inflexibility and impulsivity (r = 0.36), indicating a moderate association between these constructs. Together, psychological inflexibility and impulsivity accounted for 43% of the variance in perceived stress.

Perceived stress played a central role within the model, showing a negative association with life satisfaction (β = −0.37) and a positive association with depression (β = 0.29). Regarding explained variance, the model accounted for 33% of the variance in life satisfaction (R^2^ = 0.33) and 55% of the variance in depression (R^2^ = 0.55). In addition, the disturbance terms of life satisfaction and depression were allowed to covary, and this residual association was negative and modest in magnitude (r = −0.15), indicating that a small portion of the covariation between the two outcomes remained unexplained by the predictors included in the model. This value is not comparable to the bivariate correlation reported in Table 2 (r = −0.505), which reflects the total association before accounting for the shared predictors. The standardized path coefficients for all structural paths are presented in Figure 2.

### 3.4 Mediation analysis

Mediation analyses were conducted using bias-corrected bootstrapping with 5,000 resamples to estimate the indirect effects of psychological inflexibility and impulsivity on depressive symptoms and life satisfaction through perceived stress (see Table 3).

**Table 3:** Standardized direct, indirect, and total effects of the structural equation model.

Structural pathway	Direct effect (β)	Indirect effect (β)	Total effect (β)	95% BC bootstrap CI	*p*	Type of mediation
Psychological inflexibility → perceived stress	0.587	–	0.587	–	<0.001	Not applicable
Psychological inflexibility → depression	0.475	0.170	0.645	[0.153, 0.189]	<0.001	Partial mediation
Psychological inflexibility → life satisfaction	−0.261	−0.218	−0.479	[−0.240,−0.197]	<0.001	Partial mediation
Impulsivity → perceived stress	0.143	–	0.143	–	<0.001	Not applicable
Impulsivity → depression	0.097	0.041	0.138	[0.033, 0.050]	<0.001	Partial mediation
Impulsivity → life satisfaction	Not specified	−0.053	−0.053	[−0.065,- 0.042]	<0.001	Significant indirect effect

Standardized direct, indirect, and total effects (β) estimated from the final structural equation model. Indirect effects were estimated using bias-corrected bootstrapping with 5,000 resamples. Total effects represent the sum of direct and indirect effects when a direct path was specified. BC, bias-corrected.

For psychological inflexibility, the indirect effect on depressive symptoms through perceived stress was positive and statistically significant [β = 0.170, 95% BC CI (0.153, 0.189)]. The direct effect of psychological inflexibility on depressive symptoms remained significant (β = 0.475, *p* < 0.001), yielding a total effect of β = 0.645. Similarly, psychological inflexibility showed a significant negative indirect effect on life satisfaction through perceived stress [β = −0.218, 95% BC CI (−0.240, −0.197)]. The corresponding direct effect remained significant (β = −0.261, *p* < 0.001), resulting in a total effect of β = −0.479. These patterns are consistent with partial mediation of perceived stress in the associations of psychological inflexibility with both mental health outcomes. These direct, indirect, and total effects correspond to those reported in the revised Table 3.

For impulsivity, the indirect effect on depressive symptoms through perceived stress was positive and statistically significant [β = 0.041, 95% BC CI (0.033, 0.050)]. The direct effect of impulsivity on depressive symptoms also remained significant (β = 0.097, *p* < 0.001), yielding a total effect of β = 0.138 and supporting partial mediation. In addition, the indirect pathway from impulsivity to life satisfaction through perceived stress was negative and statistically significant [β = −0.053, 95% BC CI (−0.065, −0.042)]. This finding indicates that higher impulsivity was associated with greater perceived stress, which in turn was associated with lower life satisfaction. Because a direct path from impulsivity to life satisfaction was not specified in the final structural model, the present analysis supports a significant indirect association but does not permit a formal distinction between indirect-only and partial mediation.

Overall, perceived stress showed significant indirect effects linking psychological inflexibility and impulsivity to both depressive symptoms and life satisfaction. For psychological inflexibility, significant direct effects remained for both outcomes, supporting partial mediation. For impulsivity, partial mediation was supported for depressive symptoms, whereas the significant indirect association with life satisfaction should be interpreted cautiously because the corresponding direct path was not specified in the final model. A complete summary of the standardized direct, indirect, and total effects, together with the corresponding bias-corrected bootstrap confidence intervals, is presented in Table 3.

Taken together, the findings supported H1 and H2: psychological inflexibility and impulsivity were positively associated with perceived stress, whereas perceived stress was positively associated with depressive symptoms and negatively associated with life satisfaction. H3 was supported, as significant indirect effects through perceived stress were observed for both psychological inflexibility and impulsivity on depressive symptoms and life satisfaction. H4 was also supported: psychological inflexibility retained significant direct associations with both depressive symptoms and life satisfaction, whereas impulsivity retained a significant direct association with depressive symptoms after accounting for perceived stress.

## 4 Discussion

The present cross-sectional study examined a theoretically derived structural model integrating psychological inflexibility, impulsivity, perceived stress, depressive symptoms, and life satisfaction in Ecuadorian university students. Overall, the findings supported the proposed pattern of associations and identified perceived stress as a central transdiagnostic mechanism linking dispositional vulnerability factors with mental health outcomes. However, because of the cross-sectional design, these findings should be interpreted as evidence of statistical associations rather than causal relationships. Accordingly, the proposed mediation model represents a theoretically informed explanatory framework whose temporal ordering and causal assumptions require confirmation through longitudinal and experimental research. Within this context, psychological inflexibility emerged as the strongest vulnerability factor, showing both direct and indirect associations with depression and life satisfaction, whereas impulsivity showed smaller effects, with significant indirect associations with both outcomes through perceived stress.

From a descriptive perspective, Ecuadorian university students reported moderate levels of life satisfaction, reflecting a generally positive evaluation of their overall wellbeing despite the academic and personal challenges associated with university life. These levels were slightly lower than those reported among Portuguese adolescents (86), suggesting that life satisfaction may vary according to sociocultural, developmental, and educational contexts. This finding reinforces previous evidence indicating that life satisfaction is a key indicator of positive mental health, academic adjustment, and adaptive functioning among university students (21, 87–91). From a public mental health perspective, these results support the importance of evaluating positive wellbeing alongside indicators of psychological distress to achieve a more comprehensive understanding of mental health.

Depressive symptoms were generally low and corresponded to the mild depression category of the PHQ-9 (75), consistent with findings reported in other non-clinical university samples (92). Nevertheless, given the documented vulnerability of young adults in Latin America to mental health problems (4, 93) and Ecuador's substantial burden of depression-related disability (94), even mild depressive symptoms deserve attention. Subclinical depressive symptoms may adversely affect academic performance, interpersonal functioning, and long-term psychological wellbeing, underscoring the importance of early detection, preventive interventions, and mental health promotion strategies within higher education settings.

Students also reported relatively low levels of perceived stress, a finding that may be partly explained by the fact that data collection was conducted before the COVID-19 pandemic. This contextual circumstance likely accounts for differences from studies carried out during and after the pandemic, which consistently reported substantially higher stress levels among university students (39, 58, 95, 96). These findings underscore the importance of considering the historical context and timing of assessment when interpreting stress-related outcomes, particularly in public mental health research involving populations exposed to rapidly changing academic and social environments.

The interpretation of these findings should also consider the Ecuadorian sociocultural context. University students in Ecuador are frequently exposed to academic demands, economic constraints, and family responsibilities that may influence how stressful situations are appraised and managed (24–39). Such contextual conditions may shape perceived stress not only through exposure to daily stressors but also through culturally influenced coping strategies and emotional regulation processes. Likewise, psychological inflexibility may be influenced by social and educational contexts that affect how individuals respond to aversive internal experiences and adapt to academic challenges (48, 51–53). Although the present study was not designed to compare cultural or ethnic groups, and the sample was predominantly composed of mestizo students, previous research suggests that both perceived stress and psychological inflexibility may vary across sociocultural contexts (97, 98). Therefore, future cross-cultural studies should examine these transdiagnostic processes in more diverse Ecuadorian and Latin American populations.

Psychological inflexibility scores fell within the average non-clinical range and were comparable to normative values previously reported in Ecuadorian university students (48). However, somewhat higher levels observed in university samples from Turkey and Colombia (97, 98) suggest that sociocultural and educational contexts may contribute to differences in emotional regulation and psychological flexibility. Moderate levels of impulsivity were likewise consistent with findings from other non-clinical university populations (99, 100), supporting the conceptualization of impulsivity as a dimensional personality trait rather than a characteristic restricted to clinical conditions. Together, these findings reinforce the importance of interpreting transdiagnostic vulnerability factors within their sociocultural context rather than assuming that normative values are universally applicable.

Correlation analyses revealed significant associations among all study variables, supporting an interconnected network linking life satisfaction, depressive symptoms, perceived stress, psychological inflexibility, and impulsivity. Greater life satisfaction was consistently associated with lower levels of depression, perceived stress, psychological inflexibility, and impulsivity, reinforcing its role as a protective indicator of positive mental health in university populations (19–21). Conversely, depressive symptoms were strongly associated with perceived stress and psychological inflexibility (6, 47, 91, 101), as well as with impulsivity (54, 102), further supporting the view that depression is closely linked to stress reactivity, cognitive–emotional rigidity, and diminished self-regulatory capacity.

The structural equation model supported the proposed vulnerability–stress framework, in which psychological inflexibility and impulsivity function as dispositional vulnerability factors associated with higher perceived stress, which in turn is associated with greater depressive symptoms and lower life satisfaction. Within this framework, perceived stress emerged as a central transdiagnostic mechanism linking dispositional vulnerabilities with mental health outcomes. Psychological inflexibility showed the strongest direct and indirect associations with both depression and life satisfaction, highlighting its central role in psychological adjustment among university students. Impulsivity showed comparatively smaller effects, although significant indirect associations with both outcomes were observed through perceived stress. Because of the cross-sectional design, these findings should be interpreted as theoretically consistent patterns of association rather than evidence of causal mechanisms.

Impulsivity showed a smaller but theoretically meaningful pattern of associations within the model. Higher impulsivity was associated with greater perceived stress (β = 0.143) and showed a significant direct association with depressive symptoms (β = 0.097). In addition, perceived stress accounted for significant indirect associations between impulsivity and both depressive symptoms [β = 0.041, 95% BC CI (033, 0.050)] and life satisfaction [β = −0.053, 95% BC CI (−0.065, −0.042)]. These findings suggest that perceived stress may represent an important pathway linking impulsive tendencies not only to psychological distress but also to lower positive wellbeing. Individuals with greater impulsivity may experience difficulties in inhibitory control and self-regulation that contribute to appraising academic or personal demands as more difficult to manage, which may in turn be associated with greater depressive symptomatology and lower life satisfaction (54, 58–60). However, the magnitude of these indirect effects was substantially smaller than those observed for psychological inflexibility, suggesting that impulsivity may represent a more modest vulnerability factor within the proposed transdiagnostic framework. Because a direct path from impulsivity to life satisfaction was not specified in the final structural model, the significant indirect association with life satisfaction should not be formally classified as partial or indirect-only mediation.

Although psychological inflexibility and impulsivity were specified as independent vulnerability factors in the proposed structural model, their moderate covariance suggests that these transdiagnostic processes may operate in a complementary rather than entirely independent manner. Individuals with greater psychological inflexibility may be more likely to respond impulsively when confronted with aversive internal experiences, whereas impulsive tendencies may reduce opportunities to engage in flexible, value-consistent coping. Nevertheless, the present study was designed to estimate their independent contributions rather than to evaluate statistical interaction effects. Future longitudinal studies should examine whether psychological inflexibility and impulsivity interact synergistically to increase vulnerability to psychological distress.

These findings extend previous evidence highlighting the mediating role of perceived stress between dispositional vulnerabilities and emotional outcomes (99, 103, 104). More importantly, the present study integrates indicators of psychological distress and positive mental health within a single structural equation model while demonstrating that the magnitude of the indirect associations through perceived stress varies across vulnerability factors and mental health outcomes. Notably, the model explained substantially more variance in depressive symptoms (55%) than in life satisfaction (33%). This asymmetry is theoretically consistent with the dual-factor conceptualization of mental health (105), according to which psychological distress and wellbeing represent related but partially independent dimensions. The transdiagnostic processes examined in the present study appear to be more directly involved in the development of psychological distress than in positive evaluations of wellbeing. Accordingly, the lower proportion of explained variance for life satisfaction should not be interpreted as a limitation of the model but rather as evidence that positive mental health depends on a broader constellation of personal, interpersonal, and contextual determinants beyond those included in the present analysis.

## 5 Clinical and public health implications

From a public health perspective, the central role of perceived stress has important practical implications because stress represents a prevalent and potentially modifiable target for intervention in university populations. The present findings suggest that scalable interventions could prioritize shared transdiagnostic processes rather than addressing depressive symptoms and wellbeing separately. At the population level, universities could integrate brief stress-management, psychoeducation, psychological flexibility, and self-regulation components into existing student support and mental health promotion programs. Evidence-based approaches targeting psychological flexibility include Acceptance and Commitment Therapy (ACT), which promotes acceptance of difficult internal experiences, cognitive flexibility, and value-consistent action, whereas mindfulness-based interventions may strengthen present-moment awareness and adaptive responses to stressful experiences (106, 107). Stress-management and self-regulation programs incorporating psychoeducation and coping-skills training represent additional approaches for improving students' capacity to manage perceived academic and personal demands (108, 109). These interventions may be particularly suitable for university settings because they can be incorporated into preventive mental health programs and delivered through digital or hybrid formats, potentially increasing accessibility and scalability. Such process-based approaches are consistent with broader public mental health priorities emphasizing prevention and mental health promotion (110). Given the stronger effects observed for psychological inflexibility and the comparatively smaller effects associated with impulsivity, intervention components could also be tailored according to vulnerability profiles, with greater emphasis on psychological flexibility and stress regulation among students presenting elevated distress and additional self-regulation components when impulsivity is prominent.

## 6 Limitations and future directions

Despite its strengths, several limitations should be acknowledged. First, the use of self-report measures may have introduced response-related biases. Second, the non-probabilistic convenience sampling strategy and voluntary participation may have introduced self-selection bias, as students who chose to participate may have differed systematically from those who did not participate. In addition, the exclusion of questionnaires with incomplete responses may have introduced further selection bias. Although the final analytical sample remained large (*n* = 4,524), these sampling and participation characteristics limit the representativeness of the sample and the generalizability of the findings to the broader Ecuadorian university population.

Third, the cross-sectional design represents an important limitation for the interpretation of the mediation analyses. Although indirect effects were estimated using bootstrapping within a theoretically specified model, cross-sectional mediation cannot establish temporal precedence or causal mechanisms. Accordingly, the observed indirect pathways should be interpreted as statistical associations consistent with the proposed theoretical framework rather than evidence of causal mediation. Longitudinal and experimental studies are needed to establish the temporal ordering of psychological inflexibility, impulsivity, perceived stress, and mental health outcomes and to determine whether the proposed mediation pathways are replicated over time. This interpretation is also consistent with the methodological caution already stated earlier in the Discussion.

In addition, although participants represented different ethnic backgrounds within Ecuador, the sample was predominantly composed of mestizo students. Consequently, the present study was not designed to examine potential cultural or ethnic differences in psychological inflexibility, impulsivity, perceived stress, or their associations with mental health outcomes. Future research should include more representative and culturally diverse samples to examine whether these transdiagnostic processes and their structural pathways vary across ethnic, cultural, and educational contexts within Ecuador and other Latin American populations, thereby strengthening the external validity of the findings.

Future studies could also benefit from adopting longitudinal and process-oriented designs to examine how psychological inflexibility, impulsivity, and perceived stress unfold over time and how changes in these processes are associated with trajectories of depressive symptoms and life satisfaction. Complementing quantitative approaches with qualitative and multi-method designs may provide richer insights into stress-regulation and self-regulation processes and facilitate the translation of these findings into evidence-based public health interventions.

Finally, the BIS-8 showed moderate internal consistency in the present sample (Cronbach's α = 0.69), suggesting that findings involving impulsivity should be interpreted with appropriate caution. Although this level of reliability may be acceptable for a brief measure, measurement error may have attenuated the magnitude of some associations involving impulsivity. Future studies should therefore replicate these findings using alternative or more comprehensive measures of impulsivity.

## 7 Conclusion

This study provides empirical support for a transdiagnostic conceptualization of mental health among Ecuadorian university students, in which depressive symptoms and life satisfaction represent related but partially independent indicators of psychological functioning. By integrating psychological inflexibility, impulsivity, and perceived stress within a single structural equation model, the findings contribute to public mental health research by clarifying how shared psychological processes are associated with both distress-related and wellbeing-related outcomes in a university population.

Psychological inflexibility emerged as the strongest vulnerability factor, showing significant direct and indirect associations with both depressive symptoms and life satisfaction through perceived stress. Impulsivity showed smaller effects but was also significantly indirectly associated with both outcomes through perceived stress. Specifically, higher impulsivity was associated with greater perceived stress, which in turn was associated with greater depressive symptoms and lower life satisfaction. These findings highlight perceived stress as a relevant transdiagnostic pathway linking both psychological inflexibility and impulsivity with negative and positive indicators of mental health. However, because the direct path from impulsivity to life satisfaction was not specified in the final structural model, this finding should be interpreted as a significant indirect association rather than formally classified as partial or indirect-only mediation.

The structural model explained a substantial proportion of variance in depressive symptoms (55%) and a moderate proportion in life satisfaction (33%). This asymmetry is consistent with a dual-factor perspective of mental health, according to which psychological distress and positive wellbeing constitute related but partially independent dimensions. Accordingly, the transdiagnostic processes examined here appear to account more strongly for depressive symptoms than for positive evaluations of wellbeing, which may depend on a broader constellation of personal, interpersonal, and contextual determinants.

From a public health perspective, the findings identify perceived stress and psychological inflexibility as potentially relevant and modifiable targets for preventive and promotive interventions in higher education settings, while also suggesting a role for self-regulation processes related to impulsivity. Scalable approaches incorporating stress regulation, psychological flexibility, psychoeducation, and self-regulation components may contribute to reducing depressive symptoms while supporting positive mental health among university students.

These findings should nevertheless be interpreted in light of the study's methodological limitations. The reliance on self-report measures may have introduced response-related biases, particularly in the context of a large-scale university survey. Moreover, given the cross-sectional design, the observed indirect pathways should not be interpreted as evidence of temporal or causal effects. Longitudinal research is therefore needed to confirm the proposed temporal ordering among psychological inflexibility, impulsivity, perceived stress, and mental health outcomes and to establish the robustness of the indirect pathways identified in the present study.

## Data Availability

The raw data supporting the conclusions of this article will be made available by the authors, without undue reservation.
